# Bcl-2 interacting protein 3 (BNIP3) promotes tumor growth in breast cancer under hypoxic conditions through an autophagy-dependent pathway

**DOI:** 10.1080/21655979.2022.2036399

**Published:** 2022-02-24

**Authors:** Guipu Zhang, Zhiyi Xu, Minjing Yu, Haiyan Gao

**Affiliations:** a Department of Breast Surgery, Changzhou Cancer Hospital, Changzhou, China; b Department of Pathology, Changzhou Cancer Hospital, Changzhou, China

**Keywords:** Breast cancer, Bcl-2 interacting protein 3, BECN1, B-cell lymphoma-2, autophagy, Atg5, invasion, migration, *in vivo* and *in vitro* studies

## Abstract

Hypoxia-induced autophagy has been implicated in many cancers. Bcl-2 interacting protein 3 (BNIP3) has been associated with hypoxia, whose aberrant expression is involved in the carcinogenesis of breast cancer (BC). Here, we aim to investigate the role of hypoxia-induced autophagy and the mechanistic actions of the bioinformatically identified BNIP3 in BC. The expression pattern of BNIP3 in BC tissues and cell lines was examined using RT-qPCR and Western blot analyses. The binding affinity among BNIP3, BECN1 and BCL-2 was characterized by co-immunoprecipitation. BNIP3 expression was manipulated to assess its effects on BC cell malignant phenotypes, evaluated by cell counting kit-8, Transwell and wound healing assays, and on BC autophagy under hypoxic conditions. A BC tumor xenografts mouse model was further established to substantiate *in vitro* findings. Up-regulated expression of BNIP3 was found in BC tissues and cell lines, and BNIP3 expression was positively correlated with hypoxia exposure duration. BNIP3 knockdown restricted BC cell proliferation, invasion, and migration under hypoxic conditions. BNIP3 activated BC cell autophagy by inhibiting the binding between BCL-2 and BECN1 under hypoxic conditions. BNIP3-induced autophagy activation enhanced malignant phenotypes of BC cells, thus accelerating the tumorigenesis of BC cells *in vivo*. These data collectively supported the tumor-promoting role of BNIP3 in autophagy activation of BC under hypoxic conditions, highlighting a potential therapeutic target against BC.

## Graphical abstract

**Figure:**
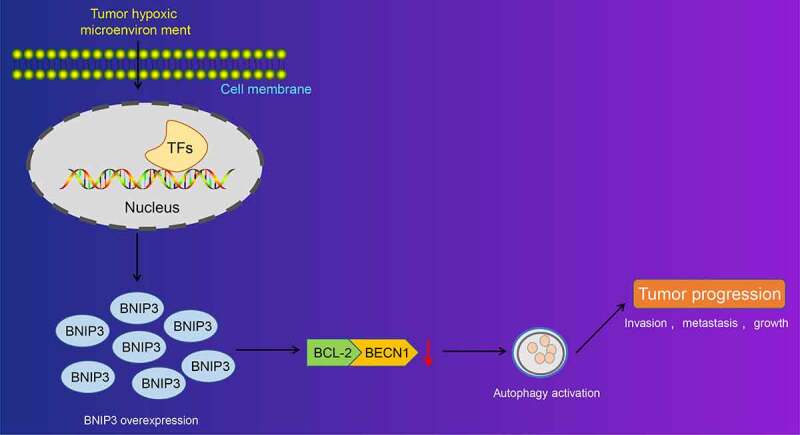

## Introduction

Breast cancer (BC) is regarded as the most prevalent malignancy as well as the leading cause of cancer-related death among females [1]. Based on both molecular and histological evidences, BC could be categorized into three subgroups: BC expressing hormone receptor (estrogen receptor (ER+) or progesterone receptor (PR+)), BC expressing human epidermal receptor 2 (HER2+) and triple-negative breast cancer (TNBC) (ER-, PR-, HER2-) [2,3]. In spite of decades of research, the incidence of BC continues to rise; nearly one in every 20 females in the world suffering from BC and one in every eight females in high-income countries suffering from BC [4]. About 70–80% of patients with early non-metastatic BC can be cured, but advanced BC with distant organ metastasis is considered incurable based on the currently available treatments [5]. Thus, it is still urgent to find reliable biochemical markers for BC diagnosis and treatment.

Hypoxia is one of the signs of cancers, which is attributed to insufficient oxygen supply, mainly due to tumor microcirculation disorders [6]. Targeting hypoxia has been considered to contribute to cancer treatment [7]. Autophagy is a lysosomal degradation pathway able to outdated proteins, remove damaged organelles and invading pathogens, showing a key role in the carcinogenesis of cancers [8]. An in-depth understanding of autophagy in the tumor microenvironment is conductive to the development of new clinical trial strategies against cancers [9].

Bcl-2 interacting protein 3 (BNIP3), a pro-apoptotic protein, is modulated by hypoxia-inducible factor 1, which has been aberrantly expressed in various cancers [10]. BNIP3 expression is related to the expression of hypoxia-regulated protein and has been demonstrated to be highly expressed under hypoxic conditions [11,12]. Up-regulation of BNIP3 has been reported to induce autophagy in cancer [13]. More importantly, findings obtained from a study suggest that BNIP3 participates in affecting the growth dynamics of BC cells [14]. But the potential mechanism of BNIP3 exerts its impacts on BC and whether it is linked to hypoxia and autophagy remains unknown. BNIP3 competes for the binding site of BECN1 on B-cell lymphoma-2 (BCL-2) through its BH3 domain, making BECN1 free so as to initiate the autophagy under hypoxic conditions [15].

Based on our findings and previous studies, we proposed that BNIP3/BECN1/BCL-2 axis could potentially stimulate BC cell autophagy so as to affect the malignancy of BC under hypoxic conditions. This study was conducted to testify this hypothesis so as to provide a better understanding of molecular mechanisms of BC and to find a new therapeutic strategy to treat BC. First, we validated the expression of BNIP3 in human BC cells and selected MCF-7 cells that presented with the highest BNIP3 expression for subsequent *in vitro* experiments. BNIP3 expression was knocked down in BC cells to assess its effects on BC cell oncogenic phenotypes under hypoxic conditions. Downstream molecular mechanisms were further explored, and a nude mouse model of BC was established to testify *in vitro* findings.

## Materials and Methods

### Ethics statements

The study was conducted under the approval of the Ethics Committee of Changzhou Cancer Hospital. All participants or their guardians signed informed consents. The experiments involving animals were performed in compliance with the recommendations in the Guide for the Care and Use of Laboratory Animals of the National Institutes of Health.

### Microarray-based gene expression analysis

BC-associated GSE45827, GSE54002 and GSE70905 expression profile were retrieved in the Gene Expression Omnibus (GEO) database. The GSE45827 expression profile included 11 normal samples and 130 BC samples. The GSE45827 expression profile included 16 normal samples and 417 BC samples, and the GSE70905 expression profile included 47 normal samples and 47 BC samples. Differentially expressed genes (DEGs) were selected in three BC-associated expression profiles using limma package in R language and identified with |*p* value < 0.05| as the screening threshold. Then, 222 autophagy-related genes (ARGs) were searched from the HADb database and intersected with DEGs to obtain differentially expressed ARGs. Kaplan–Meier plotter database was used for prognostic analysis of BC, with a threshold *p* < 0.05.

### Study subjects

BC tissues and adjacent normal tissues were collected from 30 BC patients who received surgical treatment at Changzhou Cancer Hospital from 2019 to 2020 (female/male: 30/0; age range (mean age): 28–61 (44) years old). All patients were pathologically diagnosed with BC by two experienced pathologists. None of the patients had other malignant tumors or received preoperative chemotherapy or radiotherapy. The tissue specimens obtained after resection were immediately frozen in liquid nitrogen and then stored at −80°C until further use.

### Cell culture

Human BC cell lines MCF-7, MDA-MB-468, HCC1806, and HCC1937 were purchased from KeyGEN Company (Nanjing, China). Cells were cultured in Dulbecco’s modified Eagles Medium (DMEM; GIBCO, Grand Island, NY) containing 10% fetal bovine serum (FBS) and 1% penicillin–streptomycin (HyClone, UT) at 37°C and 5% CO_2_. During hypoxic culture, cells were incubated for 48 h in a 3131 Thermo Forma Water-Jacketed CO_2_ incubator containing a mixture of 1% oxygen, 5% CO_2_ and 94% N_2_.

### Cell transfection

Transfection of MCF-7 cells with small interfering RNA (siRNA) targeting autophagy-related 5 (si-Atg5; GenePharma, Shanghai, China) was carried out according to the instruction manual of Lipo2000 (Invitrogen, Carlsbad, CA). Lentiviral hU6-MCS-CBh-gcGFP-IRES-puromycin vector containing shRNA against BNIP3 (sh-BNIP3) or Ubi-MCS-3FLAG-SV40-puromycin vector overexpressing BNIP3 (oe-BNIP3) was transduced into MCF-7 cells.

### Reverse transcription quantitative polymerase chain reaction (RT-qPCR)

Total RNA from tissues and cells was extracted using TRIzol reagent. Reverse transcription was performed with the use of a reverse transcription kit (RR047A, Takara, Japan) to generate cDNA. After the SYBR® Premix Ex TaqTM II (Perfect Real Time) kit (DRR081, Takara, Japan) was applied for sample loading, RT-qPCR analysis was performed on the ABI 7300 qPCR system (ABI, Foster City, CA). The relative expression of target genes was measured by 2^−ΔΔCt^ method, normalized to glyceraldehyde-3-phosphate dehydrogenase (GAPDH). Specific primers used are shown in Supplementary table 1.

### Western blot analysis

Total protein from tissues and cells was extracted using radio-immunoprecipitation assay lysis buffer containing phenylmethylsulfonyl fluoride, followed by lysing on ice for 30 min to collect cells. Cells were centrifuged at 8000 RPM at 4°C for 10 min to obtain supernatant. Protein concentration was detected using bicinchoninic acid protein assay kit (P0012, Beyotime, Shanghai, China). Subsequently, 50 µg of isolated proteins were dissolved in 2 × SDS loading buffer and boiled at 100°C for 10 min. Proteins were separated by 10% sodium dodecyl sulfate-polyacrylamide gel electrophoresis and transferred to polyvinylidene fluoride membranes (Merck Millipore, Billerica, MA). Membranes were blocked with 5% skimmed milk for 1 h, probed with diluted primary antibodies to BNIP3 (1:1000, #44,060, CST, MA), LC3 (1:5000, PA1-46,286, Sigma, MO), BECN1 (1:1000, ab210498, Abcam, Cambridge, UK), BCL-2 (1:1000, #15,071, CST, MA) and GAPDH (1:5000, #2118, CST, MA) overnight at 4°C, and further re-probed with the secondary goat anti-rabbit (1:20,000, ab205718) or anti-mouse (1:20,000, ab205719) immunoglobulin G (IgG) antibodies (Abcam, Cambridge, UK) for 1 h at room temperature. Electrogenerated chemiluminescence (ECL; WBKLS0100, Millipore, MA) was applied for visualization. Gray scale value of protein bands was quantified by ImageJ software (NIH).

### Co-immunoprecipitation (Co-IP)

Total protein from cells was extracted. Total 30 μL protein A agarose (Santa Cruz, CA) was added into proteins, followed by shaking at 4°C for 30 min and centrifuging to collect the supernatant. The supernatant was quantified. Total 300 μg protein was aspirated to add primary antibodies to BECN1 (1:30) or BCL-2 (1:50). The control group was shaken for 4 h at 4°C. The mixture was added 50 μL Protein A agarose and shaken overnight at 4°C and then centrifuged at 14,000 rpm for 1 min three times. The mixture was added with 60 μL 2 × loading buffer and boiled in boiling water for 5 min, followed by Western blot analysis to determine protein expression.

### Wound healing assay

The trypsinized MCF-7 cells were seeded into 6-well plates (4 × 10^5^ cells/well). Upon the cell confluence reaching 90 ~ 100%, a 10 μL disposable sterile pipette tip was adopted to create scratches. After PBS washing, fresh medium containing 10% FBS was added. The scratches were imaged 12 h later under an inverted microscope. The percentage of the area of a scratch healed by cells was analyzed by ImageJ software.

### Transwell invasion assay

Matrigel was diluted with DMEM medium at the radio of 1:8 and then spread on the chamber. The chamber was allowed to stand at room temperature for 1 h. Serum-free MCF-7 cell suspension containing 5% bovine serum albumin (BSA) was added to the upper chamber, and 500 μL of 10% FBS was added to the lower chamber. After 48 h of incubation, the upper layer of Matrigel was wiped off. Cells on the lower layer of membrane were stained with crystal violet and observed under a microscope to assess cell invasion.

### Autophagosome observation through transmission electron microscope (TEM)

MCF-7 cell suspension was seeded into 6-well plates, exposed to hypoxia and fixed in 2.5% glutaraldehyde, after which ultra-thin cell slides were prepared. Uranyl acetate was added dropwise to melted paraffin-soaked filter paper, and the grid carrying cell slides were then covered with the filter paper for 15 to 30 min to stain the cells. After another 5-to-10-min staining with lead citrate, the ultrastructural changes of cells were observed under a TEM. The double-layer membrane structure with uneven electron density in cells was considered to be an autophagosome [16].

### Immunofluorescence staining

LAMP1-mGFP and LC3-mRFP adenoviruses (GeneChem Biotechnology Company, Shanghai, China) were transduced into MCF-7 cells. After 24 h of transduction, the autoclaved cell slides were seeded into a 24-well plate with 5 × 10^4^ cells in each well. After 16 h of culture, it was observed that cells were adhered to the wall. Cells were washed twice with PBS and then fixed cells with 500 uL of 4% paraformaldehyde for 20 min. Cells were added 300 μL of 0.5% TritonX to permeabilize the cells for 10 min. After blocked with goat serum for 1 h, cells were probed with primary antibodies to LAMP1 (1:200, #9091, CST, MA) and LC3 (1:2000, #3868, CST, MA). Cells were incubated with the secondary goat anti-rabbit (#2985) or anti-mouse (#4417) IgG antibodies (Cell Signaling Technology, MA) for 1 h in the dark. About 50 μL of enzyme-labeled rabbit anti-polymer for 30 min at 37°C. Cells were cultured with 4′6-diamidino-2-phenylindole (DAPI) for 15 min. A clean glass slide was prepared, dropping anti-fluorescence quenching agent in the center. Slide with cells were placed face down in the center of the slide. Fluorescence images were taken with a Nikon confocal microscope.

### Immunohistochemistry

Fresh tumor tissue samples were cut into appropriate sizes and fixed in 4% paraformaldehyde for 24 h. After samples were dehydrated by ethanol, cells were cut into 4 um thickness and embedded in paraffin. Sections were deparaffinized, dehydrated, and treated with heat-induced antigen retrieval method in citrate repair solution on a pressure cooker. After 1.5 min of high temperature boiling, sections were cooled down at room temperature. Sections were blocked with TBS solution containing 10% normal serum and 1% BSA for 2 h at room temperature. Sections were incubated with antibodies to BNIP3 (1:100, #44,060, CST, CST, MA), LC3 (1:300, 14,600-1-AP, Proteintech, IL), while sections were treated with serum as negative control (NC) overnight at 4°C in a refrigerator. Sections were incubated with 50 μL of 3% H_2_O_2_ for 20 min at room temperature to eliminate endogenous peroxidase activity. Total 50 μL polymer enhancer was added and cultured with sections at 37°C for 20 min. Sections were re-probed with 50 μL secondary antibody (Abcam, ab205718, goat anti-rabbit, 1:2000) at 37°C for 30 min, followed by adding 2 drops or 100 μL of freshly prepared diaminobenzidine (DAB) solution. Sections were observed under a microscope for 3–10 min, and cells in brown were positive. After counterstaining with hematoxylin and dehydration, sections were sealed with neutral resin and observed under a microscope.

### Cell counting kit-8 (CCK-8) assay

MCF-7 cells were incubated in 96-well plates at the density of 5 × 10^3^ cells/well. Five parallel wells were set up in each group. After cells were exposed to hypoxia, the medium was discarded. Total 10 μL of CCK-8 reagent (Jiangsu KeyGEN BioTECH, Jiangsu, China) at 0, 12, 24, 36, 48 and 60 h was added into cells. A microplate reader was applied to measure the optical density (OD) at 450 nm. Cell viability was evaluated by GraphPad Prism 7 software.

### Reactive oxygen species (ROS) detection

Intracellular ROS was detected using a Cell Meter ROS detection kit (22,903, AAT Bioquest, Sunnyvale, CA) based on the protocols. Briefly, cells were treated with 10 μL 10× detection reagent in 96-well plates or 5 μL 5× detection reagent in 384-well plates in the presence of corresponding buffer solution, and cells treated only with buffer solution were set as the control. For the induction of ROS, ROS Brite 670 working solution was added to 96-well plates (100 μL per well) or 384-well plates (25 μL per well), followed by cell incubation for 30 to 60 min at 5% CO_2_ and 37°C. The fluorescence intensity was detected using a fluorescence microplate reader (Ex/Em = 650/675 nm).

### Tumor xenografts in nude mice

Ten BALB/c nude mice (aged 4–5 weeks, weighing about 18–22 g) were purchased from Shanghai Model Organisms Center (Shanghai, China). MCF-7 cells stably transduced with sh-NC or sh-BNIP3 were injected into the mice (n = 5). The tumor growth was observed, and the tumor volume was measured on the 21st, 24th, 27th, 30th, 33rd, and 36th days after inoculation. On the 36th day, the nude mice were euthanized by cervical dislocation. Tumor tissues were removed and tumor weight was weighed with a balance.

### Statistical analysis

The data were processed using SPSS 19.0 statistical software (IBM Corp. Armonk, NY), with *p* < 0.05 as a level of statistical significance. Measurement data were expressed as mean ± standard deviation. Data between tumor tissue and adjacent normal tissue were compared using paired *t*-test, and other data between two groups were compared by independent sample *t*-test. One-way analysis of variance (ANOVA) with Tukey’s post hoc test was adopted to analyze comparison between multiple groups. Cell viability at different time points was analyzed by two-way ANOVA while tumor volume at various time points was compared by repeated measures ANOVA.

## Results

The differential expression of BNIP3 in BC samples was first predicted through profiling of BC-related microarrays. With the aim to study the mechanistic actions of BNIP3 in BC, we determined its expression pattern in BC cells and identified the up-regulated BNIP3 expression in BC cells under hypoxic conditions. BNIP3 expression was subsequently knocked down to explore its effects on malignant phenotypes of BC cells and autophagy. Downstream mechanisms involving the autophagy-related BCL-2/BECN1 signaling were further explored in both BC cells and mouse BC xenograft model.

### BNIP3 overexpression occurs in BC tissues

**Figure 1.**
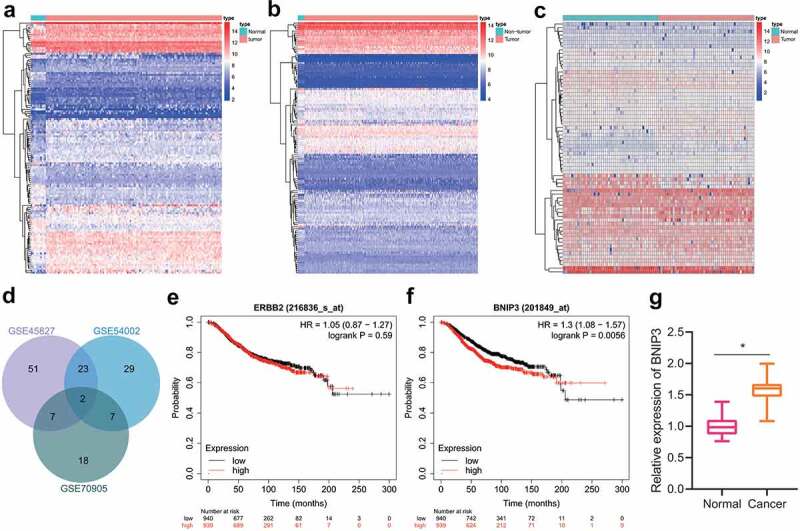
BNIP3 expression is at high levels in BC tissues. A, The expression heatmap of DGEs between 11 normal tissue samples and 130 BC samples in GSE45827 expression profile. B, The expression heatmap of DGEs between 16 normal tissue samples and 417 BC samples in GSE54002 expression profile. C, The expression heatmap of DEGs between 47 normal tissue samples and 47 BC samples in GSE70905 expression profile. D, Venn diagram showing intersections of highly expressed genes in GSE45827, GSE54002 and GSE70905 expression profiles. E, Relationship of ERBB2 with patient survival in BC prognosis. F, Association of BNIP3 with patient survival in BC prognosis. G, RT-qPCR applied for measurement of the expression of BNIP3 in 30 cases of clinically collected BC and adjacent normal tissues. Measurement data were expressed as mean ± standard deviation. Data between tumor tissue and adjacent normal tissue were compared using paired *t*-test. *p < 0.05 *vs*. adjacent normal tissues. n = 30.

Following preliminary microarray profiling, we took the intersection of highly expressed genes identified in BC samples from GSE45827, GSE54002 and GSE70905 gene-expression profiles, through which two key genes BNIP3 and ERBB2 were identified (Figure 1a–D). The Kaplan–Meier Plotter database was applied to analyze their roles in BC prognosis, and only BNIP3 was associated with poor prognosis of BC (Figure 1e and f). Meanwhile, up-regulated expression of BNIP3 has been revealed in ductal carcinoma in situ and invasive carcinoma, which suggested a regulatory role of BNIP3 in BC progression [17]. Therefore, BNIP3 was selected as a key target for BC for subsequent experiments. The results of RT-qPCR confirmed that BNIP3 was highly expressed in clinically collected BC tissues (n = 30), as compared with adjacent normal tissues (Figure 1g). The above results suggested the involvement of BNIP3 in the carcinogenesis of BC.

### BNIP3 promotes malignant phenotypes of BC cells under hypoxic conditions

**Figure 2.**
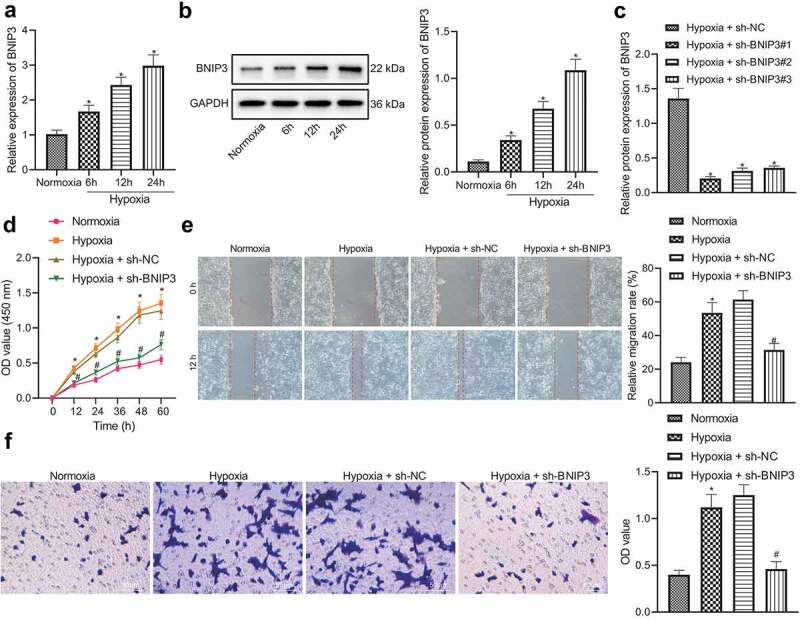
BNIP3 contributes to malignant phenotypes of BC cells under hypoxic condition. A – B, RT-qPCR (a) and Western blot (b) analysis to detect the changes of BNIP3 mRNA and protein expression in MCF-7 cells after 6, 12 and 24 h of hypoxia exposure (1% oxygen concentration) (*p < 0.05 *vs*. MCF-7 cells under normoxic conditions). C, Transduction efficiency of 3 sh-BNIP3 sequences under hypoxic conditions evaluated by Western blot analysis (*p < 0.05 *vs*. MCF-7 cells transduced with sh-NC under hypoxic conditions). D – F, Viability, migration and invasion of MCF-7 tested by CCK-8 (d), wound healing (e) and Transwell (f) assays (*p < 0.05 *vs*. MCF-7 cells under normoxic conditions; # *p* < 0.05 *vs*. MCF-7 cells transduced with sh-NC under hypoxic conditions). Measurement data from three independent cell experiments were expressed as mean ± standard deviation. One-way ANOVA with Tukey’s post hoc test was adopted to analyze comparison between multiple groups. Cell viability at different time points was analyzed by two-way ANOVA.

Hypoxic microenvironment is an important microenvironment for tumorigenesis, which is universal in solid tumors [7]. As a hypoxia-responsive protein, BNIP3 is often found to be highly expressed in cancers under hypoxic conditions [11,12]. BNIP3 expression in human BC cells (MCF-7, MDA-MB-468, HCC1806, and HCC1937) was determined to explore whether BNIP3 was involved in breast carcinogenesis under hypoxic conditions. RT-qPCR results displayed that BNIP3 expression in MCF-7 cells was the highest (Figure S1). Therefore, the MCF-7 cell line was selected for subsequent investigations. Further, BNIP3 expression was manifested to be dependent on hypoxia exposure duration: longer hypoxia exposure resulted in higher expression of BNIP3 (Figure 2a and b). Collectively, the up-regulated expression of BNIP3 might be related to the hypoxic microenvironment in BC.

Next, lentivirus carrying shRNA targeting BNIP3 was used to knock down expression of BNIP3 in MCF-7 cells under hypoxic conditions. shBNIP3#1 was selected for subsequent experiments due to its optimal knockdown efficiency (Figure 2c). Through CCK-8, Transwell and wound healing assays, it was found that relative to normoxic conditions, exposure to hypoxia could promote the proliferative, migratory and invasive potential of MCF-7 cells, which could be inhibited after down-regulating BNIP3 expression (Figure 2d–F).

The above results demonstrated that BNIP3 contributed to malignant phenotypes of BC cells under hypoxic conditions.

### Silencing of BNIP3 inactivates autophagy of BC cells by enhancing BCL-2/BECN1 under hypoxic conditions

**Figure 3.**
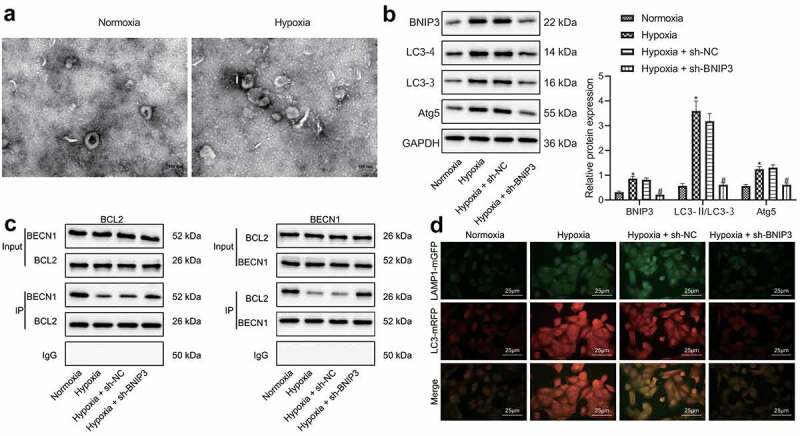
BNIP3 reduces BCL-2/BECN1 binding under hypoxic conditions to activate autophagy of BC cells. A, Autophagosomes in BC cells after hypoxia exposure observed under a TEM. B, Western blot analysis for determination of levels of autophagy-related marker proteins including LC3-II, LC3-I and Atg5. C, Co-IP assay to assesses the interaction of BECN1/BCL-2. D, Binding between LC3 and LAMP1 as detected by immunofluorescence. Measurement data from three independent cell experiments were expressed as mean ± standard deviation. One-way ANOVA with Tukey’s post hoc test was adopted to analyze comparison between multiple groups. *p < 0.05 *vs*. MCF-7 cells under normoxic conditions, ^#^*p* < 0.05 *vs*. MCF-7 cells transduced with sh-NC under hypoxic conditions.

BNIP3 played a tumor-promoting role by regulating autophagy was further explored under hypoxic conditions. Through TEM observation, it was found that the number of autophagosomes in BC cells was increased after hypoxia exposure, as reflected by increased number of bilayer membrane structures containing substances with heterogeneous electron density in cytoplasm (Figure 3a). Western blot analysis showed that compared with MCF-7 cells under normoxic conditions, the expression of BNIP3, Atg5 and LC3-II in MCF-7 cells under hypoxic conditions was elevated, the expression of LC3-I was reduced, and LC3-II/LC3-I ratio was increased, but these effects caused by hypoxia was reversed by BNIP3 knockdown (Figure 3b). The aforementioned results demonstrated that hypoxia could stimulate autophagy of BC cells, and knockdown of BNIP3 could suppress autophagy.

It has been reported that BNIP3 is an important protein that regulates the initiation of autophagy. It mainly competes with the BECN1 binding site on BCL-2 through its BH3 domain to free BECN1 and initiate the process of autophagy [15]. Consistently, Co-IP assay (Figure 3c) displayed that BCL-2 bound to BECN1 under normoxic conditions, and such binding was attenuated under hypoxic condition yet augmented when BNIP3 was knocked down. Results of immunofluorescence staining validated that hypoxia-induced MCF-7 cells presented increased LC3 level and promoted binding to lysosomal-associated protein 1 (LAMP1) in comparison with MCF-7 cells under normoxia, but additional treatment of sh-BNIP3 could abolish these roles conferred by hypoxia (Figure 3d).

The above results indicated that BNIP3 knockdown might inactivate BC cell autophagy by enhancing the binding of BCL-2/BECN1 under hypoxic conditions.

### Silencing of BNIP3 restricts malignant phenotypes of BC cells by inactivating autophagy under hypoxic conditions

**Figure 4.**
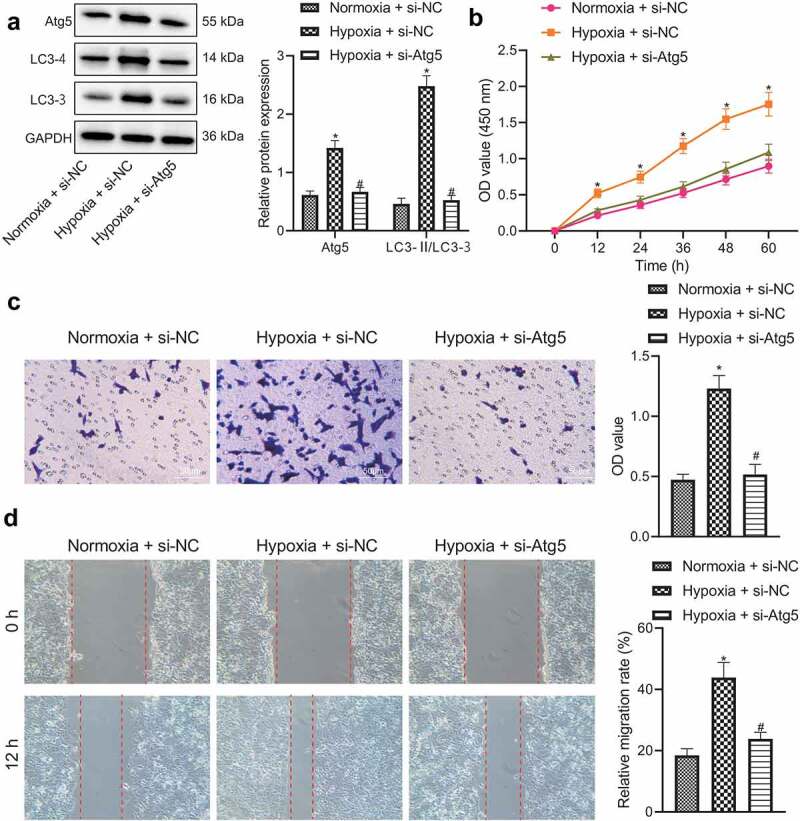
Knockdown of BNIP3 suppresses BC cell autophagy to inhibit BC cell oncogenic phenotypes under hypoxic conditions. A, Western blot analysis applied for measurement of expression of Atg5 and LC3-II in MCF-7 cells transfected with si-Atg5. B – D, Proliferation, invasion and migration ability of MCF-7 cells transfected with si-Atg5 assessed by CCK-8 (b), Transwell (c) and wound healing (d) assays. Measurement data from three independent cell experiments were expressed as mean ± standard deviation. One-way ANOVA with Tukey’s post hoc test was adopted to analyze comparison between multiple groups. Cell viability at different time points was analyzed by two-way ANOVA. *p < 0.05 *vs*. MCF-7 cells transfected with si-NC under normoxic conditions. ^#^*p* < 0.05 *vs*. MCF-7 cells transfected with si-NC under hypoxic condition.

The promoting effect of BNIP3 on BC through the activation of autophagy was further investigated. A key autophagy gene Atg5 was knocked down in BC cells. RT-qPCR was employed to evaluate the knockdown efficiency of different siRNAs, where si-Atg5#1 presented the beat knockdown efficiency and was thus selected for subsequent experiment (Figure S2). Western blot analysis results displayed that hypoxia led to elevated expression of Atg5 and the autophagy marker LC3-II and a larger radio of LC3-II/LC3-I and decreased LC3-I expression, all of which were reversed by additional Atg5 knockdown (Figure 4a). The above results validated that knockdown of Atg5 impeded BC cell autophagy under hypoxic conditions. It was further manifested that down-regulation of Atg5 could reverse the promoting effects of hypoxia exposure on BC cell viability, invasion and migration (Figure 4b–D). Taken together, it was indicated that inhibitory effect of BNIP3 silencing on BC cell malignant phenotypes under hypoxic conditions may be attributed to inactivated autophagy.

### Knockdown of BNIP3 inhibits tumorigenicity of BC cells in vivo

**Figure 5.**
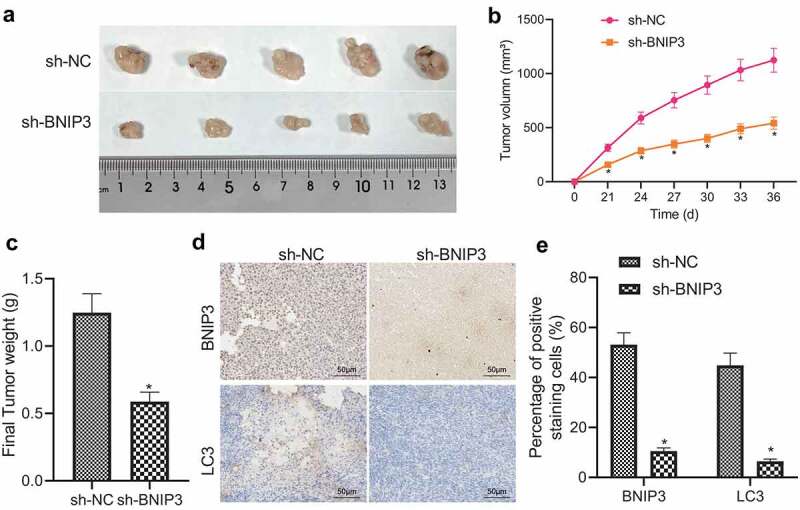
BNIP3 promotes tumorigenicity of BC cells in nude mice. A, Representative images of tumor xenografts of nude mice injected with MCF-7 cells transduced with sh-BNIP3 or sh-NC (n = 5 in each group). B, Tumor volume changes of nude mice injected with MCF-7 cells transduced with sh-BNIP3 or sh-NC (n = 5 in each group). C, Tumor weight of nude mice injected with MCF-7 cells transduced with sh-BNIP3 or sh-NC (n = 5 in each group). D – E, Representative images (d) and statistics (e) of immunohistochemical detection for expression of BNIP3 and LC3 in tumor tissues of nude mice injected with MCF-7 cells transduced with sh-BNIP3 or sh-NC (n = 5 in each group). Measurement data were expressed as mean ± standard deviation. Tumor volume at various time points was compared by repeated measures ANOVA. *p < 0.05 *vs*. nude mice injected with MCF-7 cells transduced with sh-NC.

The role of BNIP3 in BC growth was further confirmed *in vivo*. First, ROS accumulation was detected in MCF-7 cells exposed to hypoxia, which was revealed to be reduced relative to that in normoxic cells (Figure S3). MCF-7 cells transduced with sh-BNIP3 were injected subcutaneously on the right side of nude mice and those transduced with sh-NC were injected subcutaneously on the left side of nude mice. ROS accumulation in the tumor xenografts was also confirmed to be attenuated (Figure S3). Observations were conducted at day 24, 27, 30, 33, and 36 after injection, and nude mice were euthanized on the last day. It was found that the volume and weight of tumor xenografts were reduced in mice injected with sh-BNIP3-transduced MCF-7 cells relative to those injected with sh-NC-transduced MCF-7 cells (Figure 5a–C). Moreover, sh-BNIP3 resulted in diminished expression of BNIP3 and LC3 in tumor tissues of nude mice (Figure 5d–E). The above results demonstrated that knockdown of BNIP3 impeded the tumorigenic ability of BC cells *in vivo*.

## Discussion

Currently, the molecular mechanism of hypoxic microenvironment underlying the progression of BC has been increasingly focused and preliminarily explained in recent studies [18,19]. Accumulating evidence has revealed that hypoxia has favorable function on tumorigenesis and metastasis of BC [20]. Autophagy is essential for maintaining the homeostasis of cellular stress response and cell survival and has been proved to play a promoting role in BC cell survival and metastasis [21,22]. Hypoxia is able to induce accumulation of autophagosomes and increase expression of autophagy-related genes in BC cells [23]. Hypoxia-induced autophagy is associated with malignant phenotypes of BC cells has not been fully manifested. Therefore, this study mainly explored the involvement of hypoxia-induced autophagy in proliferation, invasion and migration of BC cells and its underlying molecular mechanisms. The obtained evidence revealed that BNIP3 promoted autophagy activation under hypoxic conditions so as to facilitate malignant phenotypes of BC cells.

To start with, the current study demonstrated that BNIP3 was highly expressed in MCF-7 cells and was up-regulated in response to hypoxia. Chiu *et al*. identified the up-regulated expression of BNIP3 in BC tissues and established the potent tumorigenic effect of it [24]. The tumor-initiating potential has also been reported in melanoma [25]. Contradictorily, there exists evidence suggesting the tumor-suppressing function of BNIP3 [26]. Niu *et al*. unveiled that FTO, a m6A demethylase, promoted the progression of BC through suppression of BNIP3 expression, and that BNIP3 obviously restricted FTO-dependent BC tumor growth and metastasis [14]. Given the paradoxical effects of BNIP3, its role in BC remains elusive and merits further investigations. This study illuminated that BNIP3, highly expressed in BC cells under hypoxia condition, obviously promoted the malignant behaviors of BC cells. Our findings partly corroborate a prior report where the elevated BNIP3 was uncovered to increase as the tumor progresses and upon induction of hypoxic conditions [27]. Elevation of BNIP3 expression has been noted the hypoxic regions of some solid tumors [28]. In agreement with our results, a study previously demonstrated the up-regulation of BNIP3 under hypoxic conditions in glioblastoma [29]. Ern Yu Tan *et al*. supported that BNIP3 is up-regulated in invasive cancer, which plays a key role in the transition of breast tumor progression from pre-invasive BC to invasive BC [17].

Downstream autophagy pathway related to BNIP3 was further studied, and it was revealed that BNIP3 might activate autophagy of BC cells by impeding the BECN1-Bcl-2 interaction under hypoxia, thereby contributing to BC progression. It has been reported that hypoxia could trigger autophagy accompanied by increased expression of BNIP3 [15]. BNIP3 is necessary for hypoxia-mediated autophagy, and its ability to promote the formation of autophagosomes is enhanced under conditions of nutrient deprivation [30]. Our results showed that hypoxia was able to trigger autophagy. In addition, knockdown of BNIP3 would inhibit autophagy in MCF-7 cells induced by hypoxia, as evidenced by decreased Atg5 and LC3-II but elevated expression of LC3-I in MCF-7 cells. Atg5 functions as an important participant in the progress of autophagy and regulating its expression would be a great way to modulate autophagy [31]. The conversion of LC3-I to LC3-II is also a necessary condition for the formation of autophagosomes and is widely applied as a sign of autophagy [32]. Moreover, our study demonstrated that BNIP3 activated autophagy of MCF-7 cells by inhibiting BCL-2/BECN1 under hypoxic conditions. Hypoxia-induced autophagy through BNIP3 has been validated to accelerate tumor progression [15]. Our findings revealed that BNIP3 was conductive to MCF-7 cell proliferation, invasion and migration under hypoxic conditions through increasing autophagy. Similar to our results, a previous study also emphasized the promoting role of hypoxia-associated autophagy caused by up-regulated BNIP3 on cell metastasis in cholangiocarcinoma [33]. Furthermore, the tumorigenic potential of BNIP3 was also confirmed in in nude mice.

## Conclusion

**Figure 6.**
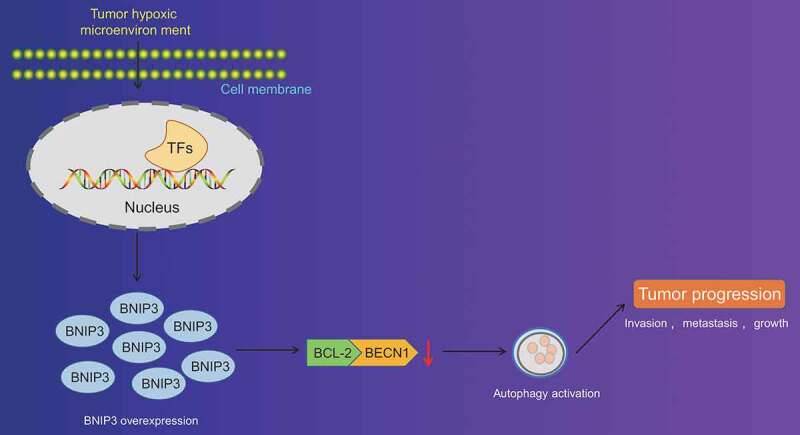
Schematic representation of the function of BNIP3 in BC cell autophagy under hypoxic conditions. BNIP3 could suppress the binding between BCL-2 and BECN1 to activate autophagy of BC cells under hypoxic conditions. Autophagy activation could enhance proliferation, invasion and migration of BC cells so as to promote the malignancy of BC.

All in all, our study collectively validated that BNIP3 could activate autophagy under hypoxic condition through inhibiting the binding between BCL-2 and BECN1 to enhance proliferation, invasion and migration of BC cells (Figure 6). By contrary, silencing of BNIP3 showed tumor-suppressing properties in BC. Therefore, the present study contributed to the identification of the role of BNIP3 in hypoxia-induced autophagy and malignant phenotypes of BC cells, offering novel therapeutic targets to inhibit BC progression. Nevertheless, current findings remain to be verified in future studies involving more BC subtypes.

## Supplementary Material

Supplemental Material

## Data Availability

The datasets generated/analyzed during the current study are available.

## Research highlights

- BNIP3 is highly expressed in response to hypoxia.
- BNIP3 enhances BC progression under hypoxic conditions.
- BNIP3 activates BC cell autophagy.
- BNIP3 activates autophagy to promote tumor growth in BC.
- This study highlights a potential therapeutic target for the treatment of BC.
