# Supplementary material for: Bcl-2 interacting protein 3 (BNIP3) promotes tumor growth in breast cancer under hypoxic conditions through an autophagy-dependent pathway
Source: Bioengineered. 2022 Feb 24;13(3):6280–92. doi: 10.1080/21655979.2022.2036399 (PMC8973668; doi:10.1080/21655979.2022.2036399)
Supplement: Supplemental Material [file KBIE_A_2036399_SM9693.zip › supplementary/Supplementary Table1.docx]

Table S1 Primer sequences for RT-qPCR

| **Gene** | **Primer sequence (5’-3’)** |
| --- | --- |
| BNIP3 | F:5’-TCCAGCCTCGGTTTCTATTT-3’ |
|  | R:5’-AGCTCTTGGAGCTACTCCGT-3’ |
| GAPDH | F:5’-GGAGCGAGATCCCTCCAAAAT-3’ |
|  | R:5’-GGCTGTTGTCATACTTCTCATGG-3’ |

### Note: RT-qPCR, reverse transcription quantitative polymerase chain reaction; BNIP3, Bcl-2 interacting protein 3; GAPDH, glyceraldehyde-3-phosphate dehydrogenase; F, forward; R, reverse.
